# QTL Genetic Mapping Study for Traits Affecting Meal Quality in Winter Oilseed Rape (*Brassica Napus* L.)

**DOI:** 10.3390/genes12081235

**Published:** 2021-08-11

**Authors:** Katarzyna Gacek, Philipp E. Bayer, Robyn Anderson, Anita A. Severn-Ellis, Joanna Wolko, Agnieszka Łopatyńska, Marcin Matuszczak, Jan Bocianowski, David Edwards, Jacqueline Batley

**Affiliations:** 1 Poznan Research Centre, Department of Genetics and Breeding of Oilseed Crop, Plant Breeding and Acclimatization Institute-National Research Institute, 60-479 Poznań, Poland; k.gacek@ihar.edu.pl (K.G.); j.wolko@ihar.edu.pl (J.W.); a.lopatynska@ihar.edu.pl (A.Ł.); marmat@nico.ihar.poznan.pl (M.M.); 2 School of Biological Sciences, University of Western Australia, Crawley, WA 6009, Australia; philipp.bayer@uwa.edu.au (P.E.B.); robyn.anderson@research.uwa.edu.au (R.A.); anita.severn-ellis@uwa.edu.au (A.A.S.-E.); dave.edwards@uwa.edu.au (D.E.); 3 Department of Mathematical and Statistical Methods, Poznań University of Life Sciences, 60-637 Poznań, Poland; jan.bocianowski@up.poznan.pl

**Keywords:** QTL mapping, candidate genes, genetic markers, rapeseed meal quality, fibre, glucosinolates, seed colour

## Abstract

Rapeseed (*Brassica napus* L.) meal is an important source of protein, but the presence of anti-nutritional compounds, such as fibre and glucosinolates, still limits its use as a livestock feed. Understanding the genetic basis of seed fibre biosynthesis would help to manipulate its content in seeds of oilseed rape. Here, we applied high-resolution skim genotyping by sequencing (SkimGBS) and characterised 187,835 single-nucleotide polymorphism (SNP) markers across a mapping population subsequently used for a genetic mapping study (R/qtl). This approach allowed the identification of 11 stable QTL related to seed quality traits and led to the identification of potential functional genes underlying these traits. Among these, key genes with a known role in carbohydrate metabolic process, cell wall, lignin, and flavonoid biosynthesis, including cellulase *GH5*, *TT10/LAC15*, *TT4*, and *SUC2*, were found. This study furthers the understanding of the molecular mechanisms underlying seed fibre content and provides new markers for molecular breeding in *B. napus*.

## 1. Introduction

*Brassica napus* L. (rapeseed, canola, oilseed rape, OSR) is grown worldwide for the production of vegetable oil, biodiesel, and protein-rich rapeseed meal (RSM) after oil extraction. A current major breeding aim in oilseed rape is to improve the quality of RSM by enhancing protein quality and quantity and reducing levels of anti-nutritional compounds, such as seed fibre and glucosinolates. The indigestible fibre present in the seed coat, including lignin, cellulose, and hemicellulose components, is the main anti-nutritional compound in black-seeded oilseed rape, which affects the taste and appearance of RSM and limits its use as a poultry feed [1]. The fibre also has a negative effect on seed oil and protein content in *B*. *napus*, as the synthesis of cellulose and hemicellulose redirects photosynthetic assimilates from oil and protein into sugar biosynthesis, which may result in reduced content of these two compounds [2,3].

Much effort has been put into the research of yellow and light-colour seeded genotypes of OSR since such genotypes have thinner seed coats, less fibre, and higher seed oil and protein content. However, these yellow-seeded genotypes have not been successfully introduced into the market due to other unfavourable agronomic traits. Seed coat colour is a difficult morphological marker for selection due to its low heritability, multiple gene inheritance, and maternal and environmental effects (light, temperature) regulating the trait [4]. Fibre, when compared to seed colour, is a more stable trait for selection [5]; therefore, understanding the genetic mechanism regulating seed fibre content is essential in improving seed oil content and meal value of *B. napus*.

Numerous studies have been performed to identify QTL for seed colour and fibre content with different effects in different genetic backgrounds. However, many of these studies revealed one major locus on chromosome A09 that explained most of the trait variation [6,7,8,9,10,11]. The QTL for fibre and seed colour are linked, as the biochemical pathways leading to the synthesis of these two economically important traits have common precursors, such as *p*-coumarate [6,12]. Although the genetic mechanism of seed fibre and colour biosynthesis have been extensively studied in Arabidopsis, the identification of their functional orthologs and understanding their mode of action in *B. napus* remains challenging. The complexity of the *B. napus* genome, which is characterised by multi-gene families, homeologous exchanges [13], and gene presence/absence variation (PAV) [14,15], further complicates the identification of key genes regulating important agronomic traits across different environments and cultivars of oilseed rape. However, whilst the latest genotyping technologies and bioinformatics allowed the identification of some candidate genes regulating fibre content and seed colour in *B. napus* [5,7,8,16], further investigations are required.

Here we performed QTL mapping analysis to unravel the genetic basis of traits affecting RSM quality, including seed oil, protein, fibre, glucosinolate, and seed coat colour using a doubled haploid (DH) mapping population derived from a cross between lines with high variation for fibre traits: yellow-seeded ‘Z114’ and black-seeded ‘M305’. The aim of the study was to identify SNPs significantly associated with the traits of interest in order to establish candidate genes and develop genetic markers that could be used in breeding programmes aimed to improve RSM.

## 2. Results

### 2.1. Phenotypic Analysis

Seed oil content (SOC), seed protein content (SPC), seed coat colour (SCC), neutral detergent fibre (NDF), acid detergent fibre (ADF), glucosinolates (GLS): 4OH-glucobrassicin (*4OH-gbs*GLS), glucobrassicin (*gbs*GLS), glucobrassicanapin (*gbn*GLS), gluconapin (*gna*GLS), napoleiferin (*nap*GLS), progroitrin (*pro*GLS), sum of alkene glucosinolates (*sum_alk*GLS) and total glucosinolate content (*sum*GLS) of the M305 × Z114 mapping population were measured in three replicates over two years of study. Extensive phenotypic variation was observed for all the traits (Table 1), but the largest variation was found for SCC, ADF, and NDF. The genotype (G), environment (E), and genotype by environment (G × E) interaction exhibited significant effects on all the traits (*p* < 0.01) (Table 1).

**Figure 1:**
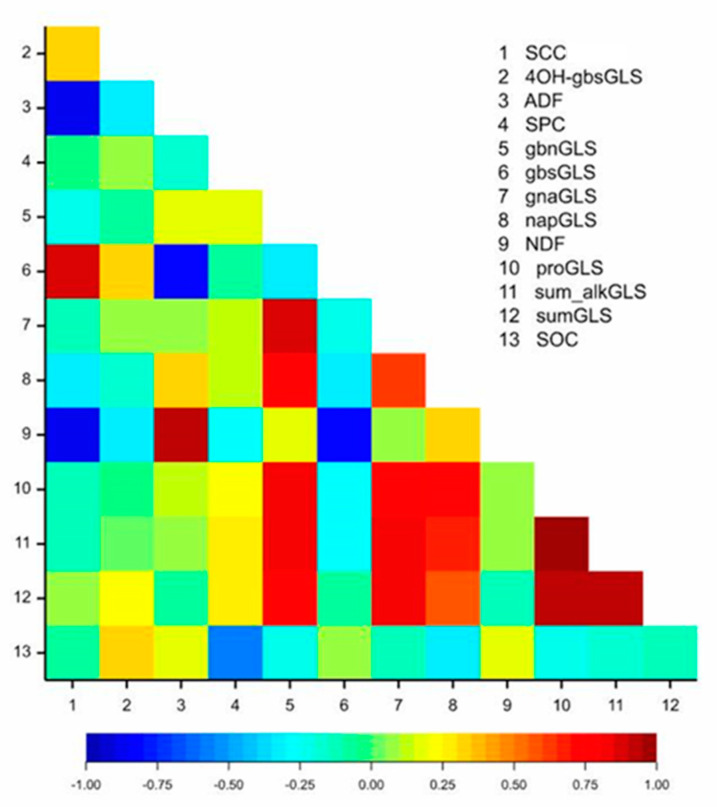
Heatmaps for linear Pearson’s correlation coefficients between the means of traits from two years of trial. (1) seed coat colour (SCC), (2) 4OH-glucobrassicin (4OH-gbsGLS), (3) acid detergent fibre (ADF), (4) seed protein content (SPC), (5) glucobrassicanapin (gbnGLS), (6) glucobrassicin (gbsGLS), (7) gluconapin (gnaGLS), (8) napoleiferin (napGLS), (9) neutral detergent fibre (NDF), (10) progroitrin (proGLS), (11) sum of alkene glucosinolates (sum_alkGLS), (12) total glucosinolates content (sumGLS), (13) seed oil content (SOC).

A strong negative correlation was observed between SCC-ADF/NDF and a weak correlation between SCC and GLS. In addition, ADF and NDF showed a weak correlation with SPC, SOC, and GLS (Figure 1). The correlations between these traits gave rise to common QTL for these traits.

### 2.2. Genome-Wide Genotyping of the Mapping Population

The sequence coverage of the parental lines was around 18× and for the DH lines, ranged from 0.89 to 3.12× (Supplementary File 1). A total of 187,835 SNP markers generated using skim genotyping by sequencing were identified between the mapping parents M305 and Z114. Of these, 146,530 could be reliably placed within the genome and used for genetic mapping. The identified SNPs were distributed across the 19 chromosomes and ranged in number from 574 on chromosome A08 to 18152 on chromosome C07, with a mean of 7712 SNPs per chromosome. For the additional unplaced contigs in the reference assembly [17], the SNP counts were between 71 on A07_random and 2888 on C01_random, with a mean of 1878 per contig (Supplementary File Table S2).

### 2.3. QTL Analysis for Seed Coat Colour, Seed Fibre, Glucosinolates, and Protein Content

To search for loci associated with seed fibre (ADF, NDF), glucosinolates (GLS), seed oil content (SOC), seed protein content (SPC), and seed coat colour (SCC), we performed genetic mapping using a population comprising 78 DH lines derived from a cross between yellow-seeded (Z114) and black-seeded (M305) parents. In total, 11 QTL were detected in both years of trial data on chromosomes A02, A04, A06, C02, C02-random, C06, C07, and C08 with a LOD score > 4 (Table 2).

QTL for GLS were detected on chromosomes A02 (position 6195793, 6195788) and A04 (position 2003804). On chromosome A04, the identified QTL included SPC (8953068) and SCC/ADF/NDF/GLS (position 18584190, 18185527). A QTL for SCC was detected on chromosome A06 (20531176). QTL for GLS were detected on chromosomes C02 (42759878) and C02-random (2378220) for GLS/SCC. Another QTL for SCC, NDF, and GLS was found on chromosome C06 (36138053) and for SCC and ADF on chromosome C07 (9412440, 9412501). On chromosome C08 (26100309), a QTL for SCC, NDF, and GLS was detected. In addition, on the same chromosome (position 35170742), a QTL for GLS was identified. Individual QTL could explain 21.52%–32.59% of the phenotypic variation (R^2^). The QTL for the other tested phenotypes (SOC, 4OH-gbsGLS) were detected only in an individual year of trial; hence, they were not analysed further (data not shown) as QTL found only in one year of a trial are not stable enough to become reliable genetic markers.

### 2.4. Candidate Gene Prediction Underling QTL for Seed Coat Colour, Seed Fibre, Glucosinolates, and Protein Content

**Figure 2:**
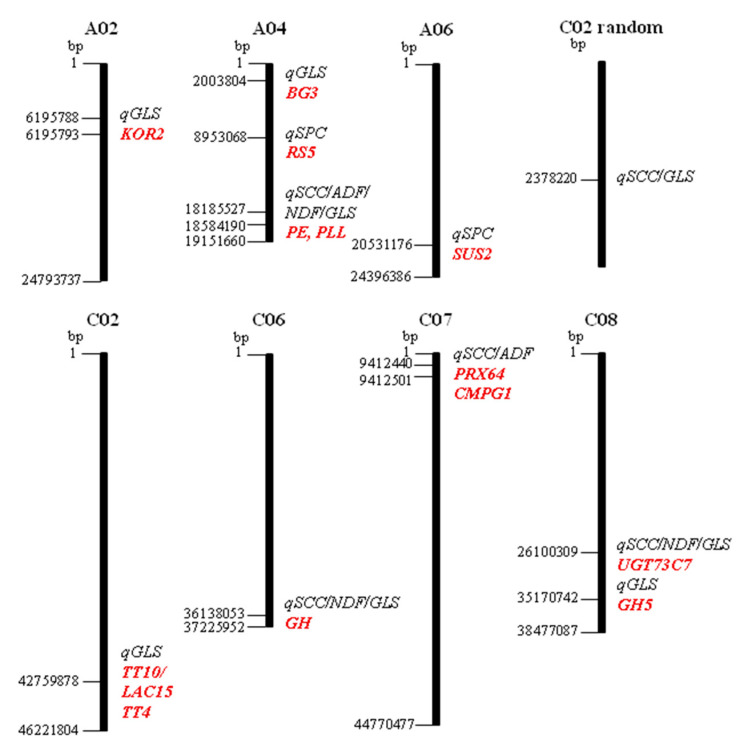
Distribution of 11 QTL associated with seed quality traits affecting RSM quality mapped onto oilseed rape chromosomes. The underlying candidate genes are highlighted in red.

To identify candidate genes that can be related to the regulation of SCC, ADF/NDF, GLS, and SPC, 11 QTL for these traits were identified in both years of field trials and analysed using the *B. napus* genome browser (http://www.genoscope.cns.fr accessed from 1 October–30 November 2020) (Figure 2).

Since the size of the detected QTL was relatively small (111 bp–405 kbp), the range of the genome screened for candidate genes was 1 Mbp upstream and downstream from the position of SNP identified as flanking the QTL in the genetic mapping study. The functional annotation of 71 possible candidate genes underlying the QTL was obtained from their homologous genes in *Arabidopsis thaliana*. A full list of genes that might be involved in the regulation of the studied traits affecting RSM quality is listed in Supplementary File Table S3 and Table 3.

Several genes underlying the QTL with a predicted role in carbohydrate metabolism, cell wall, and seed development were identified in this study. The high correlation among SPC/GLS/SCC/ADF/NDF content (Figure 1) could result from pleiotropy or close linkage between genes controlling these traits. The Z114 × M305 mapping population is contrasting the most for fibre; for this reason, many selected candidates in the QTL regions for SPC and GLS include genes involved in carbohydrate and flavonoid biosynthesis. The functional impact of nonsynonymous SNPs in these genes and how they change the encoded amino acids is shown in Table 4. In the *qGLS-A02* region, 18 candidate genes with 2–11 SNPs each were identified. The most interesting one, *BnaA02g12160D* encoding Korrigan2 (*KOR2*) that contains two missense variant SNPs, was located 7.5 kbp from the QTL. The five candidate genes underlying *qGLS-A04* contained 1–4 SNPs each, where *BnaA04g03060D,* which encodes β-1,3-glucanase 3 (*BG3*), contains three missense variant SNPs. Twenty candidate genes underlying *qSPC-A04* contained 1–9 SNPs, where *BnaA04g10260D* encodes raffinose synthase 5/seed imbibition 1-like (*RS5/ SIP1*) contained four missense SNPs. Eight candidate genes underlying *qSCC/ADF/NDF/GLS-A04* contained 1–4 SNP genetic variations. Among these, pectin methylesterase (*PE*) (*BnaA04g27070D*) is located 510 kbp from the QTL, and pectin lyase-like protein (*PLL*) (*BnaA04g25420D*) located 300 kbp from the QTL were identified. The A > C nucleotide substitution in *PE* and T > C missense variant SNPs found in the PLL gene lead to Asn/Thr and Val/Ala amino acid substitutions, respectively. The 19 genes in the region of *qSPC-A06* contained between 1 and 18 genetic variants. One of the identified candidates is sucrose synthase 2 (*SUC2*) *BnaA06g29670D* with eight missense variant SNPs and one premature stop codon. The region of *qGLS-C02* was found to have eight underlying candidate genes containing between 1 and 17 SNPs. *BnaC02g38340D* encodes transparent testa 10, laccase-like 15 (*TT10/LAC15*), and contains one missense variant. Another gene underlying this QTL, *BnaC02g38710D,* transparent testa 4 (*TT4*) encoding chalcone synthase, contains two missense SNPs. Five other candidate genes for fibre containing between 1 and 9 SNP variants were identified in the *qSCC/NDF/GLS-C06* region. One of them, encoding Glycosyl hydrolase (*GH*) (*BnaC06g38540D*), was located 59.8 kbp from the QTL and contained six missense variant SNPs. Two other candidate genes underlying *qADF/SCC-C07* include peroxidase 64 (*PRX64*) (*BnaC07g05770D*), located 173 kbp from the QTL, and RING-type E3 ubiquitin transferase (*BnaC07g05860D*) located 16.6 kbp from the QTL. Each of these genes contained two missense variant SNPs. Seven genes in *qGLS-C08* were identified with between 1 and 19 SNPs. *BnaC08g40570D,* encoding cellulase/glycosyl hydrolase family 5 (*GH5*), was located 563 kbp from *qGLS-C08* and contained 10 missense SNP variants. One of four candidate genes in *qSCC/NDF/GLS-C08,* encoding UDP-glycosyl transferase 73C7 (*UGT73C7*) (*BnaC08g24250D*), is located 177 kbp from the QTL and contains a G>A missense variant SNPs causing Ser/Asn substitution.

Identification of candidate genes associated with seed coat development, carbohydrate, and flavonoid biosynthesis in this study indicates that this approach can efficiently detect genes related to seed coat colour and fibre composition in *B. napus* seeds.

## 3. Discussion

Genotyping by sequencing allows analysis of genome-wide sequence variation among individuals, which enables accurate and efficient identification of genes controlling important agronomic traits [31].

We observed a large variation in SNP coverage between the chromosomes of the parental lines, from 574 on chromosome A08 to 15,152 on chromosome C07. Whilst A08 has the lowest number of SNPs, the short length of this chromosome makes it appears more extreme. The low marker density on chromosome A08 has already been observed in our previous study [32], and it might be due to the missing read coverage in both of the parental lines. Missing read coverage could be caused by differences between the genome of the parental lines and the Darmor reference that we used to align the reads or due to the genomic differences between those lines. The low SNP density on chromosome A08 may also be a result of a relatively low level of genetic diversity between the parental lines as they both represent low erucic acid and low glucosinolates (double zero) winter-type oilseed rape [33]. Intensive breeding of double zero oilseed rape led to a restricted gene pool, which reduces its genetic variation. Chromosome A08 could also represent a genomic region of identity-by-descent that was not efficiently disrupted by recombination during selection [34].

Seed fibre and glucosinolates considerably reduce the value of *B. napus* meal, especially for poultry; therefore, the identification of functional candidate genes related to these traits is of importance [35,36,37,38]. In the studied population, derived from the yellow-seeded ‘Z114’ and black-seeded ‘M305’ DH lines, the highest phenotypic variation was found for fibre (ADF/NDF) and SCC. These traits were correlated with glucosinolates and protein content in previous studies [39]. Decreased amounts of cell wall polysaccharides in seeds containing less fibre can cause increased carbon availability for protein deposition [40].

The correlation found between ADF/NDF/SCC/GLS/SPC can be reflected in the identification of common QTL for the studied traits (e.g., qSCC/ADF/NDF/GLS) and many interesting candidate genes related to plant cell wall, lignin biosynthesis underlying SPC and GLS-QTLs. The QTL for these traits was found on chromosomes A02, A04, A06, C02, C06, C07, and C08, with PVE ranging between 21.52% and 31.65%. The regions of A02, A04, A06, C02, C06, and C08 were also found to be correlated with seed fibre by Miao [11]. It is difficult to compare the positions of the identified QTL regions due to different reference genomes used by Miao (ZS11) and here in this study (Darmor). QTL for ADF and GLS were found previously on chromosome C02 [10], whereas A06, C08, and A09 regions were repeatedly detected for SCC in a GWAS study performed by Wang ^4^. Here we found a similar region of A06 (20.5 Mbp) and C08 (distal end) to that found in the Wang GWAS study. Interestingly, the distal region of chromosome C08 correlated with SCC, NDF, and GLS in our study showed high homology with a region of chromosome A09 [4,13], detected as a major QTL for seed fibre and seed colour in various genetic backgrounds [2,5,6,7,8,9,17]. Major QTL for seed colour were also detected on A09 or C08 chromosomes, depending on the genetic background [41], which also indicates that different black-seeded forms may possess different seed colour genes. Previous studies also showed a correlation with seed fibre on chromosomes A05 and C05; however, here, we did not detect any QTL on these chromosomes [3,10].

A number of candidate genes associated with seed fibre deposition, seed coat development, flavonoid, and anthocyanin biosynthesis were identified in previous studies [1,4,16]. Strong candidates include cinnamoyl-CoA reductase 1 (*CCR1*) and cinnamyl alcohol dehydrogenase (*CAD2/CAD3*), *SEC8*, *PAL4*, *CESA3*, and *GPAT5* [2,3,5,7,8]. Some of the candidate genes identified in this study belong to the same gene family but are located on a different chromosome. The most interesting candidate genes *BnaC02g38340D* and *BnaC02g38710D* were identified here for seed fibre, and SCC was located on chromosome C02 and encoded transparent testa 10/laccase-like 15 *(TT10/LAC15)* and transparent testa 4 (*TT4*), respectively. In other studies, *TT4* was found to be associated with ADL (acid detergent lignin) on chromosome C09 (*BnaC09g43250*) [3], and *TT10* was detected as a major gene for SCC and fibre on A09 [5,9]. Transparent testa (*TT*) are key enzymes in proanthocyanidins and lignin biosynthesis pathways [9,25,28,42].

Since the highest phenotypic variation in the Z114 × M305 mapping population was found for ADF/NDF and SCC, the strongest emphasis on the identification of candidate genes was made for genes known to be involved in carbohydrate metabolism and flavonoid biosynthesis. In the *qGLS-A02* region, one of the identified genes, Korrigan2 (*KOR2*), encodes endo-1,4-β-d-glucanase, known to be involved in cellulose synthesis [18,43]. Interestingly, the same region of chromosome A02 was found to be correlated with seed fibre in a study performed by Miao [11]. The interesting candidate genes underlying QTL regions on chromosome A04 include pectin methylesterase (*BnaA04g27070D*) and pectin lyase-like protein (*BnaA04g25420D*). The presence of SNP variation in these genes and their relatively close physical locations from the QTL (100–500 kbp) indicates that they are very likely regulators of fibre composition in *B. napus* seeds. Another gene, *BnaA04g03060D,* located 10 kbp from *qGLS-A04*, encodes β-1,3-glucanase 3, glycoside hydrolase, which functions in cell wall degradation [19]. Other interesting genes underlying QTL on chromosome C08 include cellulase, glycosyl hydrolase family 5 *GH5,* and UDP-glycosyl transferase *UGT73C7,* which are known to be involved in carbohydrate metabolic process and cell wall lignification [27,30]. Another strong candidate is peroxidase 64 (*PRX64*) (*BnaC07g05860D*), located 173 kbp from the *qSCC/ADF-C07,* the major oxidase enzyme known to play a role in proanthocyanidins and lignin biosynthesis [9,25,28,43]. RING-type E3 ubiquitin transferase (*CMPG1*) (*BnaC07g05860D*) with two SNPs located 16,6 kbp from the QTL. These genes are known to play a role in lignin biosynthesis and response to chitin [21,29,44]. A key gene found to be correlated with seed colour in previous studies, namely transparent testa 12 (*TT12*), was not identified in this study. *TT12* encodes a multidrug and toxic compound extrusion (MATE) secondary transporter that is specifically expressed in the developing seed coat and is involved in the transportation of proanthocyanidin precursors into the vacuole [45]. It was found that the *BnaC06g17050D* gene, which is orthologous to Arabidopsis *TT12*, is associated with seed coat colour in oilseed rape [4]. However, we could not find any association between this gene and SCC in the present study.

## 4. Materials and Methods

### 4.1. Plant Material and Field Trials

The experimental population (M305 × Z114) consisted of 78 doubled haploid (DH) lines, developed from F_1_ plants of a cross between single plants: M305, a black-seeded *B. napus* DH line, and Z114, a yellow-seeded *B. napus* DH line. The two parental lines were selected for contrasting seed coat colour and seed fibre content.

The yellow-seeded lines originated from an interspecific cross between a *B. napus* natural mutant with brighter seeds (double low quality) identified in PBAI-NRI and a *B. napus* spring line obtained from Canada Agriculture Research Station with segregating seed coat colour (seeds with yellow dots). The spring line from Canada originated from a cross between *B. napus* × *B. rapa* [46].

The M305 × Z114 mapping population was cultivated in a PBAI-NRI experimental field in Poznan, Poland, during two growing seasons: 2015/2016 and 2017/2018. The field trials were carried out in a randomised block design with three replicates of double 3 m-long rows. The field was managed with standard methods. Five plants per genotype were bagged to ensure self-pollination and threshed for further analysis.

### 4.2. Trait Analysis

The contents of seed oil, protein, fibre (acid detergent fibre, ADF and neutral detergent fibre, NDF), glucosinolates (GLS): 4-OH glucobrassicin, glucobrassicin, glucobrassicanapin, gluconapin, napoleiferin, progoitrin, and total GLS were determined in the Laboratory of Biochemistry PBAI-NRI in Poznan using a near-infrared reflectance spectroscopy (NIRS)—Infratec 1255 analyser. The measurements were averaged over three replicates per line per year. The quantification of seed coat colour was determined with The Hunter Labs spectrocolorimeter (Colorflex, VA, USA) and classified using a 0 (black) to 5 (yellow) scale [47]. The relationships between the traits were assessed based on Pearson’s correlation coefficients and tested with the *t*-test.

### 4.3. Genotyping and SNP Marker Analysis

Genomic DNA (gDNA) from the individual plants within the lines was extracted using a standard Doyle/CTAB method and prepared for genotyping by sequencing. The DNA was quantified using a Qubit 3.0 Fluorometer with the Qubit dsDNA BR Assay Kit (Invitrogen, Carlsbad, CA, USA), and the quality was assessed using the LabChip GX Touch 24 (PerkinElmer, Waltham, MA, USA). A total of 200 ng of DNA was taken from each sample for shearing on the M220 focused-ultrasonicator system (Covaris, Woburn, MA, USA). DNA libraries with an insert size of 550 bp were prepared for sequencing according to the manufacturer’s protocol using the TruSeq Nano DNA Library Prep kit (Illumina^®^, San Diego, CA, USA). Before pooling, the completed libraries were quantified using a Qubit, and the quality was examined using a LabChip (GX Touch 24, PerkinElmer Waltham, MA USA). Whole-genome sequencing (100 bp, PE) was performed at the Australian Genome Research Facility (AGRF) in Melbourne (Victoria, Australia) (Illumina^®^ HiSeq, Illumina^®^, San Diego, CA, USA).

Reads were aligned to the *B. napus* Darmor v4.1 reference genome using soap v2.21 (paired read settings, allowing an insert size between 0 and 1000 bp, reporting only uniquely aligning reads) [48], and converted to bam files using samtools v1.6 [49]. The parental reads were merged using samtools, and duplicate reads were removed using Picards’s MarkDuplicates (http://broadinstitute.github.io/picard/ accessed on 1 August 2020). SNPs were called per chromosome using SGSautoSNP using default settings [50]. Progeny genotypes were called using the SkimGBS pipeline [51].

### 4.4. QTL Mapping

QTLs were mapped using R/qtl v1.44-9 [52]. All data were loaded using cross-type double-haploid (‘dh’), and genotype probabilities were calculated with an error probability of 0.01 (error.prob = 0.01). A single QTL model was run using Haley–Knott regression (method = ‘hk’). *p*-value cutoffs were determined using 1000 permutations.

### 4.5. Identification of Candidate Genes

Physical mapping of SNPs significantly correlated in the genetic mapping study, and functional annotation of the predicted genes was performed using the *B. napus* genome browser (http://www.genoscope.cns.fr/brassicanapus/) [17]. The Arabidopsis Information Portal (Araport) https://www.araport.org/, The Arabidopsis Information Resource (TAIR) https://www.arabidopsis.org/, EMBL-EBI https://www.ebi.ac.uk/, Ensembl Plants http://plants.ensembl.org/index.html, and Kyoto Encyclopedia of Genes and Genome (KEGG) (http://www.genome.jp/kegg/genes.html) databases were used to identify genes that play a role in carbohydrate, flavonoid, glucosinolates and protein metabolism. The database websites were visited between 1 October 2020–30 November 2020. The percentage of variance explained (PVE) was calculated using the formula PVE=1−10−2·LODn.

## 5. Conclusions

In conclusion, a QTL genetic mapping study using an NGS SkimGBS approach allowed us to identify several promising genes, including PE, PLL, TT10/LAC15, SUS2, and GH5, which provides insight into the complex genetic architecture of seed fibre and colour biosynthesis in B. napus. Understanding the mechanism of action and causal polymorphisms of these genes will provide a better understanding of the role of those genes in the regulation of complex traits affecting RSM quality.

## Figures and Tables

**Table 1:** Phenotypic variation of the yellow-seeded (Z114) and the black-seed (M305) parents and the M305 × Z114 DH population from two years of trials.

Trait	Z114 Mean	M305 Mean	DH Mean	DH Range		E	G	G×E
					d.f.	1	79	79
SOC	41.6 ± 3.1	43.4 ± 2.06	41.5 ± 3	35.45–49.46		***	***	***
SPC	24.21 ± 1.95	24.3 ± 1.3	25.05 ± 1.4	19.33–29.36		***	***	***
SCC	4.70 ± 0.6	0.47 ± 0.65	1.54 ± 0.75	0.03–5.1		***	***	***
NDF	17.13 ± 0.85	23.05 ± 0.9	21.36 ± 1.9	16.47–26.86		ns	***	***
ADF	10.05 ± 0.7	16.9 ± 0.8	14.88 ± 2.4	6.79–22.15		***	***	***
*4OH-gbs*GLS	4.96 ± 0.45	4.52 ± 0.6	4.98 ± 0.7	2.1–6.82		***	***	***
*gbs*GLS	0.36 ± 0.03	0.21 ± 0.01	0.23 ± 0.04	0.12–0.4		***	***	***
*gbn*GLS	0.33 ± 0.15	0.47 ± 0.1	0.57 ± 0.2	0.03–2.15		***	***	***
*gna*GLS	1.23 ± 0.5	3.88 ± 0.6	2.59 ± 0.9	0.54–9.04		***	***	***
*nap*GLS	0.08 ± 0.01	0.01 ± 0.01	0.10 ± 0.02	0.04–0.25		**	***	**
*pro*GLS	1.91 ± 1.19	2.73 ± 1.55	4.18 ± 2.2	0.1–20.9		***	***	***
*sum_alk*GLS	3.75 ± 2.65	5.19 ± 2.27	7.49 ± 3.46	0.12–30.47		***	***	***
*sum*GLS	9.22 ± 2.05	8.96 ± 2.1	12.13 ± 3.5	4.47–31.23		***	***	***

Seed oil content in % (SOC), seed protein content in % (SPC), seed coat colour (SCC), neutral detergent fibre in % (NDF), acid detergent fibre in % (ADF), glucosinolates (GLS) in µmol g-1: 4OH-glucobrassicin (4OH-gbsGLS), glucobrassicin (gbsGLS), glucobrassicanapin (gbnGLS), gluconapin (gnaGLS), napoleiferin (napGLS), progroitrin (proGLS), sum of alkene glucosinolates (sum_alkGLS), total glucosinolates content (sumGLS). ** *p* < 0.01; *** *p* < 0.001; ns–not significant.

**Table 2:** The stable QTL detected in both years of field trials for traits affecting RSM quality in the Z114 × M305 mapping population.

QTL	Trait	Chr	Position	LOD Score	R^2^ (%) PVE
*qGLS-A02*	*sum*GLS, *gna*GLS, *nap*GLS, *gbn*GLS *pro*GLS, *sum_alk*GLS	A02	6195793, 6195788	4.27, 6.61	21.79, 31.65
*qGLS-A04*	*pro*GLS, *nap*GLS	A04	2003804	6.55	31.41
*qSPC-A04*	SPC	A04	8953068	5.52	27.22
*qSCC/ADF/NDF/GLS-A04*	SCC, ADF, NDF, *gbs*GLS	A04	18584190, 18185527	4.32, 4.21	22.02, 21.52
*qSPC-A06*	SPC	A06	20531176	4.49	22.78
*qGLS-C02*	*gbn*GLS, *pro*GLS	C02	42759878	6.85	32.59
*qSCC/GLS-C02r*	SCC, *gbs*GLS	C02-random	2378220	4.62	23.35
*qSCC/NDF/GLS-C06*	SCC, NDF, *gbs*GLS	C06	36138053	4.82	24.23
*qSCC/ADF-C07*	SCC, ADF	C07	9412440, 9412501	4.78, 5.59	24.05, 27.51
*qSCC/NDF/GLS-C08*	SCC, NDF, *gbs*GLS	C08	26100309	4.42	22.46
*qGLS-C08*	*gbn*GLS, *sum*GLS, *sum_alk*GLS	C08	35170742	4.77	24.01

Seed protein content (SPC), seed coat colour (SCC), neutral detergent fibre (NDF), acid detergent fibre (ADF), glucosinolates (GLS), glucobrassicin (gbsGLS), glucobrassicanapin (gbnGLS), gluconapin (gnaGLS), napoleiferin (napGLS), progroitrin (proGLS), sum of alkene glucosinolates (sum_alkGLS), total glucosinolates content (sumGLS).

**Table 3:** Candidate genes underlying 11 stable QTL.

QTL	QTL Size	No. Genes	*B. napus*	*A.thaliana*	Functional Annotation	References
*qGLS-A02*	2.5 kbp	20	BnaA02g12160D	AT1G65610.1	Korrigan 2 *(KOR2)*	[18]
*qGLS-A04*	4,8 kbp	5	BnaA04g03060D	AT3G57240.1	β-1,3-glucanase 3 (*BG3*), glycoside hydrolase, cell wall degradation,	[19]
*qSPC-A04*	70.3 kbp	20	BnaA04g10260D	AT5G40390	Raffinose synthase 5 *(RS5)*, Seed imbibition 1-like *(SIP1)*	[20]
*qSCC/ADF/NDF/* *GLS-A04*	405.8 kbp	8	BnaA04g27070D BnaA04g25420D	AT2G47550.1 AT2G43860.1	Pectin methylesterase, (*PE*) cell wall modification Pectin lyase-like protein, (*PLL)*, carbohydrate metabolic process	[21,22]
*qSPC-A06*	1.9 kbp	19	BnaA06g29670D	AT5G49190	Sucrose synthase 2 (*SUS2*) seed maturation, starch, sucrose metabolic process, plant cell wall	[23,24]
*qGLS-C02*	111 bp	8	BnaC02g38340D BnaC02g38710D	AT5G48100.1 AT5G13930.1	Transparent testa 10, laccase-like 15, (*TT10/LAC15*) lignin and flavonoids biosynthesis Transparent testa 4, (*TT4*) chalcone synthase, flavonoid biosynthesis	[25,26]
*qSCC/GLS-C02r*	1 kbp	1	BnaC02g47290D	AT5G46040.1	Major facilitator superfamily protein	
*qSCC/NDF/GLS-C06*	575 bp	5	BnaC06g38540D	AT1G78060.1	Glycosyl hydrolase, (*GH*) carbohydrate metabolic process, plant-type cell wall	[27]
*qSCC/ADF-C07*	240 bp	2	BnaC07g05770D BnaC07g05860D	AT5G42180.1 AT5G01830.1	Peroxidase 64, (*PRX64*), lignin biosynthesis, plant cell wall RING-type E3 ubiquitin transferase; (*CMPG1*), response to chitin	[28,29]
*qSCC/NDF/GLS-C08*	380 bp	4	BnaC08g24250D	AT3G53160.1	UDP-glucosyl transferase 73C7 (*UGT73C7*)	[30]
*qGLS-C08*	308 bp	7	BnaC08g40570D	AT1G13130.1	Cellulase, glycosyl hydrolase family 5, (*GH5)*, carbohydrate metabolic process	[27]

**Table 4:** The effect of SNPs identified in candidate genes regulating seed ADF/NDF content.

QTL	*B. napus*	SNP Position	Alleles	Effect of SNP
*qGLS-A02*	BnaA02g12160D	6406888 6407629	G>T A>G	missense Val>Phe missense Arg>Gly
*qGLS-A04*	BnaA04g03060D	2014574 2014706 2014718	C>A A>G T>G	missense Pro>Gln missense Glu>Gly missense Leu>Arg
*qSPC-A04*	BnaA04g10260D	9043708 9045761 9046164 9046782	T>C C>T A>T C>A	missense Tyr>His missense Thr>Ile missense Lys>Asn missense Phe>Leu
*qSCC/ADF/NDF/* *GLS-A04*	BnaA04g27070D BnaA04g25420D	19096076 18283611	A>C T>C	missense Asn>Thr missense Val>Ala
*qSPC-A06*	BnaA06g29670D	20209248 20209490 20209679 20209685 20210405 20210426 20210576 20210746 20212314 20212875	C>T A>C C>T C>T A>G C>T G>A A>C A>C G>A	missense Ser>Leu missense Ile>Leu missense Arg>Cys missense Pro>Ser missense Arg>Gly missense Pro>Ser missense Glu>Lys missense Arg>Ser stop missense Gly>Asp
*qGLS-C02*	BnaC02g38340D BnaC02g38710D	41321282 41652670 41652738	T>A G>C C>G	missense Phe>Ile missense Trp>Ser missense His>Asp
*qSCC/GLS-C02r*	BnaC02g47290D	3064584 3064753 3064912 3066441	T>A T>A G>C T>C	missense Ser>Thr missense Leu>Gln stop missense Phe>Leu
*qSCC/NDF/GLS-C06*	BnaC06g38540D	36074371 36074734 36075755 36075812 36076103 36078104	A>G C>T A>G C>A C>G C>T	missense Gln>Arg missense Thr>Ile missense Thr>Ala missense Leu>Ile missense His>Asp missense Arg>Trp
*qSCC/ADF-C07*	BnaC07g05770D BnaC07g05860D	9237994 9238393 9428459 9428556	A>T G>A C>T G>A	missense Gln>Leu missense Arg>His missense Ala>Val missense Val>Ile
*qSCC/NDF/GLS-C08*	BnaC08g24250D	26289421	G>A	missense Ser>Asn
*qGLS-C08*	BnaC08g40570D	35735275 35735343 35735471 35735528 35735805 35735820 35736579 35736883 35737048 35737117	T>A G>C A>G C>T C>T A>T T>C T>G C>G T>G	missense Ser>Arg missense Glu>Gln missense Asp>Gly missense Ala>Val missense Pro>Ser missense Ile>Leu missense Tyr>His missense Ile>Met missense Ile>Met missense Asn>Lys

## Data Availability

Read data presented in this study are openly available at the SRA under PRJNA727897.

## Supplementary Materials

The following are available online at https://www.mdpi.com/article/10.3390/genes12081235/s1, Supplementary File 1, Table S1: Sequencing coverage for the parental lines and individual DH lines; Supplementary File 2, Table S2: Number of SNPs identified using the standard SkimGBS protocol, with the Darmor v4.1 reference genome Supplementary File 3, Table S3: Candidate genes underlying 11 stable QTL.
